# Supplementary material for: Identification of photoperiod-induced specific miRNAs in the adrenal glands of Sunite sheep (Ovis aries)
Source: Front Vet Sci. 2022 Jul 22;9:888207. doi: 10.3389/fvets.2022.888207 (PMC9354845; doi:10.3389/fvets.2022.888207)
Supplement: Supplementary file 1 [file Data_Sheet_1.ZIP › Supplementary file/miRDeep2 file.zip!/miRDeep2 file/Know_Structure/SP42A3/oar-mir-655.pdf]

miRBase precursor : oar-mir-655
Total read count : 1729
oar-miR-655-5p read count : 1
oar-miR-655-3p read count : 1728
remaining reads : 0

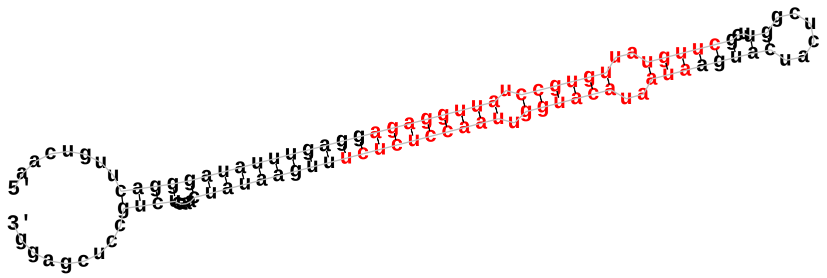

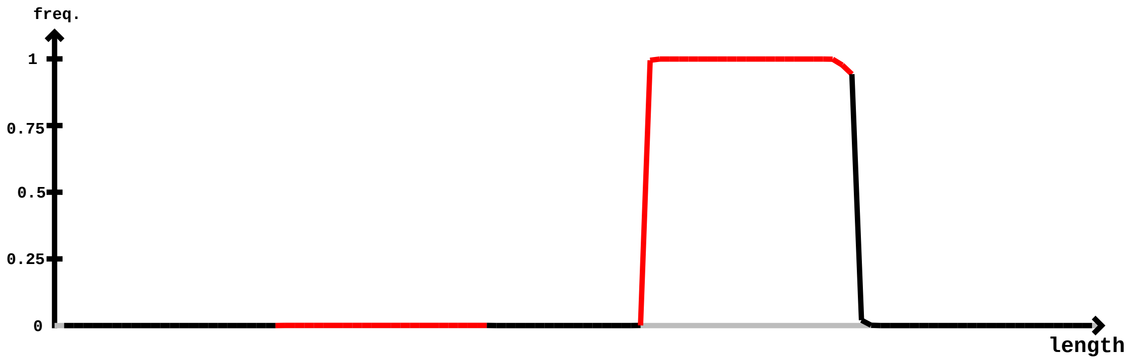

oar-miR-655-5p

oar-miR-655-3p

```
5'- aacuguucagggauauuugaggagagguuauccguguuauguucgcuuggcucaucaugaauaauacaugguuaaccucucuuugaauaucaaacucugccucgagg   -3'    exp
    ..........(((((((((((((((((((.((((((..((((((..((......))))))))..)))))).)))))))))))))))))))...(((......))).   reads     mm        sample
    ......................agagguuauccguguuauguuc...............................................................   1         0         seq
    ...........................................................aauaauacaugguuaaccuc............................   1         0         seq
    ............................................................auaauacaugguuaaccucu...........................   38        0         seq
    ............................................................auaauacaugguuaaccucuc..........................   59        0         seq
    ............................................................auaauacaugguuaaccucucu.........................   1587      0         seq
    ............................................................auaauacaugguuaaccucucuu........................   33        0         seq
    ............................................................auaauacaugguuaaccucucuuu.......................   2         0         seq
    .............................................................uaauacaugguuaaccucucu........................   8         0         seq
```
